# Influence of Anatomy on the March of Civilization

**Published:** 1858-02

**Authors:** J. C. Nott

**Affiliations:** Professor of Anatomy in the University of Louisivna


### SELECTIONS.

From the N. O. Medical and Surgical Journal.

*Influence of Anatomy on the March of Civilization*: By J. C. Nott M. D., Professor of Anatomy in the University of Louisivna.

The science of medicine is so widely separated from the range of thought of the great mass of mankind, (even of those who have been liberally educated) that few, out of our profession, have any proper appreciation of the intimate connection which exists between its various branches and other departments of human knowladge, and a still smaller number have any idea of the immense influence the medical profession has exercised on the march of civilization. Could the people and their legislators be made fully sensible of the truths I propose to set forth, they would not hesitate to award to medical colleges, the credit which they justly merit. Few States in the Union have fostered these institutions with such liberal hands as that of Louisiana, and yet her people are already feeling benefits which will increase from year to year. But my object now is not simply to show that we are elevating the standard of medical education from year to year, and supplying the population with more intelligent and more skilful physicians, but that while we place the science of medicine on a more elevated platform, we, at the same time, elevate all other departments of knowledge with it. As my space will not permit allusion to other departments of medical education, as chemistry, botany, etc., I shall confine my illustrations simply to that of Anatomy, to which too little importance is attached by those outside of our profession, and I might add with truth, by too many in the profession.

So legitimately are all the departments of human knowledge connected together, that it becomes very difficult to estimate the full value of any one, or its influence on others, however disconnected they may seem. It may, however, be safely asserted that there exists no science, or no department of knowledge so isolated, that it does not receive light direct or indirect from others.

It may be laid down as an axiom, that *all knowledge is derived from the study of nature.* The mind of man comes into the world a blank, and would remain so were it cut off from the observation and study of objects of nature. Locke, in his immortal work on the understanding was the first to develope fully the great truth that all knowledge comes through our senses, for though the mind in its progress may develope certain abstract ideas, even these, if closely anaylzed, will be found to have their ground work in ideas received through the senses. Imagine a man who had never *seen* an object in nature—who had never heard a sound—who had never felt, smelt or tasted, and ask what abstract ideas his mind could originate, that would possess form or value. In fact, all our knowledge is the result of the action of our minds on materials derived from observation of the objects and phenomena of the external world. The boasted mind of man can *invent* nothing but *falsehood*—all science—all knowledge is but the discovery of facts of nature and their relations.—What, for example, is the noble science of astronomy? It is no *invention* of man. The master minds of Newton, Kepler, Laplace. Herschel, Leverier and others, have but *discovered* a few of the laws which have governed the celestial bodies for millions of years. So with mathematics, natural philosophy, botany, zoology, geology, chemistry, anatomy, etc.

Every proposition in Euclid is based on a law of nature. It was not Euclid that made the right angle of a triangle equal to the sum of the other two angles—or the square of the hypothenuse equal to the sum of the squares of the other two sides. When the chemist tells us that all substances in nature may be resolved into some sixty simple elements—that these may be recombined to form well known inorganic compounds; and that they combine in certain definite proportions, he only proclaims *discoveries* of beautiful laws of nature.

How stands the case with our own science of medicine? What are we daily striving at? Is it not simply to extend our *discoveries?* We seek with the dissecting knife and microscope to extend our knowledge of the structure of the human body. We seek to extend our knowledge of its functions, and the action of morbid causes upon it; and we seek in the vegetable and mineral kingdoms for remedies to heal its maladies. Every new splint the surgeon shapes, or new instrument he brings to light, is but an appliance to carry out a mechanical principle which exists in nature.

We may then, I think, fairly assume the truth of the two propositions thus briefly alluded to: First, That the mind of man commences its existence in darkness, and would remain so, were it not for the perceptions received through the five senses, which place us in relation with the external world. Second, All our knowledge is derived from the observation of nature, and the operation of our minds upon ideas so obtained.

If, then, it be true that the mind of man is enlightened and expanded by the study of nature, it is evident that the wide field covered by the science of anatomy must yield the richest of all harvests, and one well worthy of the great minds of Cuvier, Palas, Blainville, Geoffroy, St. Hilaire, Agassiz, John Hunter, Owen, Leidy, and many others, who have not only widely extended the horizon of speculative, but of useful knowledge.

You must not commit the grave error of thinking that the science of anatomy belongs to the doctor's shop alone. On the contrary, anatomy in its more general and philosophic acceptation, is the *Science of Organization*, considered in the different living beings, from the simplest zoophyte up to man. The series of organized beings forms an immense chain, the extent of which the accumulated labors of ages has not been able to measure. The air, the earth, the mighty deep are all populated by living beings, which, by their infinite variety of organization, form and size, attest the inexhaustible fecundity of nature. Thus while among the mammiffers life is maintained by the most complicated apparatus, we find at the other end of the scale, animals, like the hydra, polyps, etc., which have been alternately ranged with animals and vegetables. Nor are the varieties in size less striking, ranging from the mammoth or whale down to the animalculæ, a million of which the microscope discovers in a single drop of water. Even the animalculæ, so infinitely small to our senses, may in turn become gigantic, compared to others yet to be discovered with more perfect instruments.

You at once perceive then that anatomy is the most vast of all sciences—far too vast for any one mind. Hence the necessity of dividing it up into many departments, any one of which may fully occupy the lifetime of one man.

We shall proceed to point out in succession, the connection between anatomy and a few of the more prominent departments of human knowledge, viz: mental philosophy, fine arts, zoology, geology, jurisprudence, political economy, ethnology, natural and revealed religion, etc.

1st. Let us inquire what connection exists between anatomy and mental philosophy? Science is one thing—religion is another, and should in the present sensitive state of the public mind, be kept as much apart as possible, and it is not our desire here to make any allusion to the soul, or immortal part of man; but the *mind*, the *intellectual powers*, are clearly functions of that organ called the brain, and we should commence our study of the mind, by the *anatomical* structure of the organ from which it emanates. The mind is almost null in the new born infant—it grows and gains strength on to mature age—it is deranged by disease of the brain, and is annihilated by compression and other injuries. Every thing, then, proves that its intellectual functions depend upon the integrity of the anatomical structure of the *brain*.

The metaphysician who attempts to investigate thought, and to decompose the complicated operations of the understanding, can make no solid progress without laying his ground work in the structure, and functions of the various parts of the brain, organs of sense, and spinal system. The leading mental philosophers of the past age were fully sensible of the importance of anatomy, and Descartes, Loche, Malebranche, Condillac and others, were versed in anatomy and took it as their point of departure. But it is to the labors of Gall, and those who have followed in his footsteps, that we are mainly indebted for our advance in mental philosophy. While on the one hand, I do not mean to say that the present state of our knowledge justifies us in mapping out the faculties of the brain on its surface, as Gall has done, still the merit cannot be denied him, of having given a new impetus and better direction to the study of mental philosophy. He has at least clearly proven, that certain grand divisions of the brain, preside over certain groups of functions—the anterior—the posterior parts of the brain—the cerebellum—and the medulla oblongata; although their functions are not all clearly defined, yet differ widely in those they perform. And not less important are the great discoveries of Sir Charles Bell, of the functions of the spinal marrow, and its system of nerves. A familiar illustration of all this may be given in the organs of sense—the eye—the ear—olfactory organ—the tongue, have each a nerve leading to and connected with a point of the brain, and each has a distinct function to perform; did the optic nerve connect with the brain at the point of origin of the auditory nerve, there would be no vision, and *vice versa* of the auditory nerve; it is clear, then, that one point of the brain gives sight, another hearing, another smell, another taste, etc., showing clearly that the brain is a compound organ, having many functions to perform.

And here comes in the great science of comparative anatomy, to show how indispensable it is to the proper understanding of man, and to illustrate the remark of Buffon, that if "no other animal than man had lived, we should know much less of man than we do.

In fact, most of our knowledge of the functions of the nervous system of man, has been derived from the dissections of animals. Now man belongs to that division of the animal kingdom to which the term *vertebrata* has been assigned by naturalists. The leading characteristic of this division is found in the existence of a brain, spinal marrow, and vertebral column. The vertebrata have been subdivided into mammifers, birds, reptiles and fishes, all of whom have a brain and spinal marrow.

Anatomy teaches us that man possesses the most complicated and perfect nervous system known, and by following the chain on downwards through the mammalia, birds, reptiles and fishes, we find that the brain becomes smaller and less perfect, one organ after another is diminished or left out, until in the lowest links but little brain and little intelligence is left. If we pass on from the *Vertebrata*, through the three remaining divisions of the animal kingdom, viz: *Articulata*, the *Mollusca*, and the *Radiata*, we shall find that not only the brain, but the entire nervous system disappear, and even the special organs of sense.

It is by anatomy, then, that the mental philosopher has been able to study and analyze the structure and functions of the nervous system. He can go back and commence almost at the worm, and by following on the chain upwards through fishes, reptiles, insects, and warm-blooded animals, discover that one organ after another is added, each of which has its special use, until he reaches such structures as the brains of Napoleon, Newton and Locke.

I have pointed out to you one of the great results of comparative anatomy. It is the ground work of mental philosophy, and no intellect, however gigantic, could ever trace the connection between mind and matter, without the dissecting knife and microscope.

Even sculpture and painting, those twin arts, that so charm the eye, refine the taste, and expand the mind of man, are not less indebted to the aid of anatomy for the high perfection to which they have attained in our day. A certain amount of anatomical instruction is now considered as essential in every school of design. What excellence could the historical painter attain, without a knowledge of those muscles of the face which give expression to the various passions and emotions of the mind ; or of those of the limbs and trunk which give expression to the motions and attitudes of living man? The most beautiful object in nature to us, is the human form divine ; who can gaze upon the Apollo or the Venus, the Laocoon, or the dying Gladiator, without the most pleasurable emotion; and they give us pleasure because they are true to nature. It may be said th t the sculptors who produced these unrivalled works of art, lived in a day when dissections were not tolerated, and anatomy was in its infancy. In reply, we say, that we do not know with certainty even the names of many of the artists, and therefore can know little of their knowledge of anatomy; but we can say, whether they did or did not dissect, they studied the external anatomy of man with a fidelity which never can be surpassed; the swell and outline of every muscle, the eminences and depressions of every bone, the motions of every joint, every trivial furrow, every superficial blood vessel, or tendon, the irregularities of the skull, etc., were all depicted with a fidelity which can never be excelled. No one, then, will deny that anatomy is an essential element in the education of the sculptor and painter.

Need I now address myself to the intelligent jurist and ask him to turn to Paris and Fontblanque, Beck, Taylor, and other works on Medical Jurisprudence, and say how much he owes to the labor of the anatomist, to say nothing of medical chemistry? How often is the anatomist called before the coroner, or into courts of justice to determine a point of life and death. Questions of infanticide have often to be decided by the testimony of the medical man alone; in cases of death arising from blows, wounds, etc., it is the anatomist who is to decide, and upon his testimony alone, hangs, in many instances, the lives of human beings, and the momentous decisions of damning guilt or of innocence. But it would be almost an insult to intelligence to dwell upon a point so palpable as this.

Where shall I begin, or where shall I end, when I undertake to tell you of the connection between anatomy and the vast science of *Zoology?* What is meant by the term *Zoology?* It covers the entire physical history of every living being, and every fossil remain, from the animalculæ or zoophyte up to the most gigantic animal forms. Zoology, then, must necessarily have comparative anatomy for its ground work. Comparative anatomy not only examines into the minute structure of each organized species, but it is its province to compare them together, to study their affinities or discordances, to arrange them into groups having anatomical relation, and to open to our view that stupendous plan by which an All-wise Providence has populated and repopulated our globe. We have only to turn back to the works of Buffon to prove how indispensable is anatomy to the science of zoology. Buffon was a truly great and useful man in his way, and to the charm which his genius threw around Natural History, are we greatly indebted for the impetus given to its study during the past century. No man was ever more deeply impressed with the grandeur and beauty of Nature's works; no man ever searched more diligently for landmarks by which to classify and arrange the immense mass of material over which his mind wandered; and never did gorgeous and attractive style do more in awakening the attention of others to the study of Nature's works; but after all there was something wanting to give solidity and durability to his plan, and to elevate Natural History to the level of a true science. It was the want of *anatomy.*

When the naturalist of our day looks over the fifty volumes of Boffon, he marvels at the extent of his labor; is fascinated with his beautiful descriptions of the external forms of animals, their habits, instincts, geographical distribution, etc.; but when we seek for those solid materials out of which to construct an exact science, we turn to the labors of Daubenton, the modest, but gifted collaborateur of Buffon, who has left us dissections of nearly two hundred species of animals, so full and accurate as to leave nothing to be desired, as far as these details extended; but Daubenton attempted no complete classification of the animal kingdom.

Comparative anatomy, by leading us to an exact appreciation of the organic resemblances and differences presented by all animals, is now regarded as the sole solid basis of their classification.

It is to Cuvier that we are indebted for the first truly scientific classification of the animal kingdom, which is founded on the organization, and the principle of natural anatomical affinities. It follows the descending order, beginning with the most complex type at the summit, and placing the most simple at the bottom of the scale. He divides all known animals into two grand anatomical divisions, viz: the *Vertebrata*, or those having a spinal column, and second, the *Invertebrata*, or those having no spinal column. The latter are again divided into *Articulata*, *Mollusca*, and *Radiata*, and the whole animal kingdom is thus divided into four grand departments, which are based entirely on anatomical distinctions. If we go on down through the orders, classes, families, generations and species, we find that anatomy is still our guide, and the only means by which we can surely place each animal. It is only when well marked anatomical distinctions cease, that our doubts and disputes commence.

When, for example, you place beside each other, a bear and a dog, you discover anatomical differences which at once point to specific difference and distinct origin. But when you place beside each other the dog, wolf, fox and jackal, though they have some external differences, their anatomical structure is identical—they have the same number and form of teeth, the same number and form of bones in the skeleton, and the same internal organs; but, the comparative anatomist requires but a single bone or tooth of the bear to say that it is not a dog, or a wolf, or deer—but a bear, and nothing but a bar.

If the question be asked, "Are all horses of one species or origin?—are all dogs—all cattle—all our domestic sheep and goats of one species—nay, more, are all the races of men of one species? We are at sea without a compass. Anatomy ceases to guide us, and it is probable tnat such disputes will never be settled.

And what shall I say of the noble science of Geology, which strives to know the chronology and formation of the globe? It goes hand in hand with comparative anatomy, like sculpture with painting—in fact, organic remains are the hand-writing of the Almighty upon stone, by which he has revealed to the geologist all that we know of Preadamite history of the Earth, and it is to the anatomical labor of the immortal Cuvier, that we are indebted for our most important geological knowledge.

The following quotations, from the work of Professor Hitchcock, on "Geology and Religion," in allusion to the fossil remains of extinct animals, will give some idea of the intimate and extensive connection between comparative anatomy and geology. "The fossiliferous rocks," says he, "or such as contain animals and plants, are not less than *six or seven miles in perpendicular thickness*, and are composed of hundreds of alternating layers of different kinds, all of which appear to have been deposited, just as rocks are now forming, at the bottom of lakes and seas; and hence their deposition must have occupied an immense period of time.—Even if we admit that this deposition went on in particular places much faster than at present, a variety of facts forbids the supposition that this was the general mode of their formation.

"The remains of animals and plants found in the earth are not mingled confusedly together, but are found arranged, for the most part, in as much order as the drawers of a well regulated cabinet. In general, they appear to have lived and died on or near the spots where they are now found; and as countless millions of these remains are often found piled together, so as to form almost entire mountains, the periods requisite for their formation must have been immensely long.

"Still further confirmation of the same important principle, is found in the well established fact, that there have been upon the globe, *previous to the existing races, not less than five distinct periods of organized existence;* that is, five great groups of animals and plants, so completely independent, that no species whatever is found in more than one of them, have lived and successively passed away, before the creation of the races that now occupy the surface. Other standard writers make the number of these periods of existence as many as twelve. Comparative anatomy testifies, that so unlike in structure were these different groups, that they could not have coexisted in the same climate and other exernal circumstances.

"There are abundant facts to prove that the climate of the whole globe was warm or even warmer than the tropics of our day, and the slow change from warmer to colder, appears to have been the chief cause of the successive destruction of the different races; and new ones were created, better adapted to the altered condition of the globe; and yet each group seems to have occupied the globe through a period of great length, so that we here have another evidence of the vast cycles of time that must have rolled away even since the earth became habitable.

"Among the thirty thousand species of animals and plants found in the rocks, very few living species have been detected; and even those few occur in the most recent rocks, while in the secondary group, not less than six miles thick, not a single species now on the globe has been discovered· Hence the present races did not exist till after those in the secondary rocks had died."

Another very curious result of anatomical investigations is the fact, "*that the laws of anatomy have always been the same since organic structures began to exist.*"

"It has long been known that the organs of animals were beautifully adapted to perform the functions for which they were intended. But it was not till the investigations of Baron Cuvier, within the last half century that it was known how mathematically exact is the *relation* between the different parts of the animal frame, nor how precise are the laws of variation in the different species, by which they are fitted to different elements, climates, and food. It is now well known that each animal structure contains a perfect system of *correllation*, and yet the whole forms a harmonious part of the entire animal system of the globe.

To use the language of Cuvier: "Every organized individual," says he, "forms an entire system of its own, all parts of which mutually correspond, and concur to produce a certain definite purpose, by reciprocal action, or by combining towards the same end. Hence, none of these separate parts can change their forms without a corresponding change in the other parts of the same animal, and consequently each of these parts to which it has belonged. Thus, if the viscera of any animal are so organized as only to be fitted for the digestion of recent flesh, it is also requisite that the jaws should be so constrccted as to fit them for devouring prey; the claws must be constructed for seizing and tearing it to pieces; the teeth for cutting and dividing its flesh; the entire system of the limbs, or organs of motion, for pursuing and overtaking it; and the organs of sense for discovering it at a distance. Nature must also have endowed the brain of the animal with sufficient instinct for concealing itself, and laying plans to catch its necessary victims." This is the picture of a carnivorous animal, and the same adaptation of structure and instincts to mode of life will be found in the herbiverous and all others, large or small, perfectly or imperfectly organized. "Hence," says Cuvier, "any one who observes merely the print of a cloven hoof, may conclude that it has been left by a ruminant animal, and regard the conclusion as equally certain with any other in physics or in morals. Consequently, the single foot mark clearly indicates to the observer the forms of the teeth, of all the leg bones, thighs, shoulders, and of the trunk of the body of the animal which left the mark.

"By thus employing the method of observation, where theory is no longer able to direct our views, we procure astonishing results. The *smallest fragment of bone*, even the most apparently insignificant apophysis, posseses a fixed and determinate character relative to the class, order or genus and species of the animal to which it belonged; insomuch, that when we find merely the extremity of a well preserved bone, we are able, by a careful examination, assisted by analogy and exact comparison, to determine the species to which it once belonged, as certainly as if we had the whole animal before us.

Cuvier first fully tested this law of correllation of parts, in existing animals, but it remained for him to apply the same principles to remains of fossil animals. It was certain that if the laws of anatomical structure were the same in the pre-Adamite creations, as they are now, the above principles must apply equally well to the bones found in the rocks, and though often only scattered fragments are brought to light, the anatomist would be able to *reconstruct* the whole animal, and present him to our view.

The quarries aroundParis had furnished a vast number of bones of strange animals, and these had been thrown promiscuously into the collections of the city. Cuvier determined here to put his principles to the test amidst this huge and confused mass of fossil remains. "I found myself," says he, "as if placed in a charnel house, surrounded by mutilated fragments of many hundred skeletons, of more than twenty kinds of animals, piled confusedly around me. The task assigned me was to restore them all to their original position. At the command of *comparative anatomy*, every bone, and every fragment of a bone resumed its place. I cannot find words to express the pleasure I experienced in seeing, as I discovered one character, how all the consequences which I predicted from it were successively confirmed; the feet were found in accordance with the characters announced by the teeth; the teeth in harmony with those indicated beforehand by the feet; the bones of the legs and thighs, and every connecting portion of the extremities, were found to set together precisely as I had arranged them, before my conjectures were verified by the discovery of the parts entire; in short, each species was, as it were, reconstructed from a single one of its component elements."

Here, then, we have demonstrated, by the light of anatomy, that the same laws of life which now prevail in the world, have existed through a succession of creations, through thousands, tens of thousands, and probably millions of years.

Organic remains, too, teach us that there have been, in the remotest geological epochs, viviparous as well as oviparous creatures, and gemmiparous as well as fissiparous animals and plants. They were also carnivorous, herbivorous, and omniverous animals; they posses the same internal organs as animals of our day and they were governed by the same physiologibal laws as those of the present day.

What a wonderful achievement it is for anatomy, that it should thus have exhausted the existing Fauna of the earth, and have f llowed up, step by step, the foot-prints of the Almighty, through this plan so vast in time, so gigantic in extent, and so infinite in wisdom!

Nor is anatomy without intimate connections with both natural and revealed religion. Of the latter, I have not time or inclination to speak here; but what need is there of volumes on natural religion with facts before us like those I have presented. No sane man could follow this long chain of organized beings through creation after creation, through those countless ages of time, could reflect on that plan which has been working with such harmony from beginning to end, and doubt there is a Supreme and Overruling Intelligence, and that we are responsible to him, and him alone, for our acts in this world.

Had we time, I could easily show you to what extent even physics are indebted to anatomy. The human frame is a beautiful machine, from which the mechanic arts have received many useful lessons. It is a system of levers, fulcrums, pulleys, admirably arranged for motion and strength. The celebrated Kipler devoted much study to the structure of the eye. It suggested to the mind of Euler the possibility of achromatic lenses, and the whole science of optics has gained much from the study of the eye. Of like importance has been the anatomy of the ear, on which is based the science of acoustics.

I will conclude by calling your attention for a few moments, to one point more, and not the least important, viz: the connection between anatomy and ethnology.

When we cast our eyes over the broad surface of the globe, and behold the endless variety of human beings which inhabit it, of every shade of complexion, of the most opposite physical conformation, and of the most diverse moral and intellectual characters; I say, when we look upon all this, in spite of the influence of early education, we are driven to ask ourselves the question, "Are these races of men of one parentage, and are they alike capable of moral and intellectual cultivation?" The *origin of man* is a momentous question, one which has elicited much discussion among the wisest heads, and is one which I shall not touch here; but still the physical history of man has certain important connections with the view of anatomy I am now placing before you, which render it impossible for me to pass it by in silence, without doing manifest injustice to the science of anatomy, which it is my duty to teach.

Of the precise epoch at which man was placed on the earth, we know nothing. The short chronology of Archbishop Usher, of six thousand years, is now abandoned by every well informed writer in the church and out of the church; it may be ten thousand, twenty thousand, or one hundred thousand for ought we know. Nor can we divine how far back such well marked races as Jews, and other white races; Negroes, Mongols, Hottentots, Australians, Indians, Malays, etc., etc., first made their appearance. This much, however, has been positively demonstrated through evidence derived from Egyptian, Assyrian, and Chinese monuments and records, viz., that the Jews and other white races, as well as Negroes, Egyptians, Mongols and other well marked types of man existed at least five thousand years ago as distinct from each other as they now are; and it is equally certain that these forms have, during this long period, been permanent and unchang[illegible]d by climate or other existing physical causes; no race during this time has been transformed into another, whatever miracles may have been perf[illegible]rmed in earlier ages

We not only have evidence of the existence of distinct races from the Bible, Homer, Herodotus and other early writings, but we have actual portraits of races, on the monuments of Nineveh, Babylon, and Egypt, dating back several thousand years. Nay, more, if all this were not sufficient, we have other overwhelming evidence of the antiquity of races.—We have the mummies of Negroes, Jews, and other races taken from the ancient catacombs of Egypt, which place the question at once in the hands of the anatomist. We have other human remains from the barrows of Britain and Germany, from the mounds of America, from the ancient burying grounds of Mexico and Peru, from the ruins of Nineveh, from the bone caverns and other sources, all of which give to the anatomist ample material for proving the *permanency* of types through all known physical influences, and all known time

When these different races of men are brought together and compared *anatomically* they are found to differ quite as widely in structure, as do the species in most of the genera of animals. Compare, for example, the skeletons of the African Negro, and the Anglo-Saxon, and I do not hesitate to assert that they differ as much as those of the polar and tropical bears—the horse and the ass—the dog and the jackal, or the lion and tiger; but we shall call your attention to but one anatomical point by way of illustration. I will select the brain, as it is the organ of intelligence, which should form the most striking and satisfactory point of distinction.

The lamented Dr. Morton, of Philadelphia, who has done more in this department of anatomy than any other man, collected and measured accurately more than one thousand skulls of various races. Since his death, his collection, which has been deposited in the Academy of Sciences, of Philadelphia, has been considerably extended, and has been re-measured by Dr. Meigs, with the same results. It has thus been ascertained that not only do the races show a great variety of forms in the external configuration of the skull, but a marked difference in the size of the brain. If we compare together the two extremes, viz., the Teutonic and North European races below the Arctic, with the Hottentot of the Cape of Good Hope and the Australian, we find the former have seventeen cubic inches more of brain than the latter; if then we take the Mongol races, the Malays, the Negroes of tropical Africa, the American Indians, and other races, we shall find that there exists a gradation between the two extremes, and that anatomy assigns to each its proper position in the scale.

Nor is it less certain that the large-headed, fair-skinned races have been the rulers of the world and the depositories of all true civilization. The Mongol races, who have brains intermediate in size between the white and black races, have long since achieved a semi-civilization beyond which they cannot rise; while the intellects of the darker races have remained as dark as their complexions. From the Barbary States to the Cape, no race has ever invented an alphabet—no manuscript or tablets have been found to tell their rude history, and no architectual remain has been found to mark a bright spot on the five thousand years, during which we know they have existed.

Now, *when* did this great inequality of the races begin? Of this we know nothing, but the great practical fact remains that it has existed for several thousand years, and we have every reason to believe that it will remain till these races are extinct. We have shown that both anatomy and history prove that the physical cause now in operation on earth has not been able to transform one type into another.

There are well meaning philanthropists who talk to us about the influence of education and sing hosannahs over the wonderful results which are flowing from the colony of Liberia, and the efforts of Missionaries in civilizing these inferior races. Anatomy teaches us that it is impossible, as history has before done, until you can fall upon some plan by which seventeen cubic inches of brain can be added to that of the Hottentot, the Bushman, or the Australian, you cannot add to his intellect, or his capacity for civilization. I have examined this question with a great deal of care, and can find no facts, or evidence whatever to show that education can enlarge the brain. We know that the muscles of the arm of the blacksmith will enlarge by the constant use of the hammer, but when did thought ever increase the size of the brain, any more than vision does that of the eye, or hearing the nerve of the ear? There is no analogy between the actions and functions of the nervous system, and those of the muscular system.

The brains of royal families—of nobility, which have been educated classes for centuries, have no larger heads than the lower classes. Educated communities have no advantage in this respect, over the uneducated. The ancient Britons and Germans had the same skulls as their more civilized descendants. The heads of Napoleon, Newton, Shakespeare and Locke were as large at the age of sixteen as at fifty. In a word, there is no more evidence to prove that you can change the size and form of head in a race, than that you can change the form of a negroe's foot, his pelvis, his thick lips and nose, his black skin and wooly hair. These are anatomical peculiarities stamped on him by his maker at some time unknown to us, and nothing short of the miraculous interference of the same power can change bis anatomical structure. Anatomy thus teaches us a great practical fact and one which should be well understood, by the statesman the philanthropist and the theologian. We must take the works of God as we find them, and not suffer ourselves to be misguided by Utopian schemes. As Knox quaintly said, you cannot change an Irishman into an Englishman by act of parliament.

But it is time that I should draw this rambling essay to a close. The subject is sufficiently extensive for volumes, and if I have succeeded in directing thought to the value of anatomical studies my aim is fully met

It must be clear to any one who takes the trouble to investigate the subject, that anatomy has, from the time of Aristotle to the present day, exercised a controlling influence over many departments of human knowledge, over the progress of civilization.

Read the history of the world and you will find that medical colleges have ever been the foci around which have clustered the whole circle of natural sciences.

